# The impact of post-fall huddles on repeat fall rates and perceptions of safety culture: a quasi-experimental evaluation of a patient safety demonstration project

**DOI:** 10.1186/s12913-019-4453-y

**Published:** 2019-09-09

**Authors:** Katherine J. Jones, John Crowe, Joseph A. Allen, Anne M. Skinner, Robin High, Victoria Kennel, Roni Reiter-Palmon

**Affiliations:** 1 College of Allied Health Professions, University of Nebraska Medical Center, 984420 Nebraska Medical Center, Omaha, NE 68198-4420 USA; 2 Department of Psychology, University of Nebraska at Omaha, 6001 Dodge Street, Omaha, NE 68182-0274 USA; 3 College of Public Health, University of Nebraska Medical Center, 984375 Nebraska Medical Center, Omaha, NE 68198-4375 USA

**Keywords:** Post-fall huddles, Teamwork, Safety culture

## Abstract

**Background:**

Conducting post-fall huddles is considered an integral component of a fall-risk-reduction program. However, there is no evidence linking post-fall huddles to patient outcomes or perceptions of teamwork and safety culture. The purpose of this study is to determine associations between conducting post-fall huddles and repeat fall rates and between post-fall huddle participation and perceptions of teamwork and safety culture.

**Methods:**

During a two-year demonstration project, we developed a system for 16 small rural hospitals to report, benchmark, and learn from fall events, and we trained them to conduct post-fall huddles. To calculate a hospital’s repeat fall rate, we divided the total number of falls reported by the hospital by the number of unique medical record numbers associated with each fall. We used Spearman correlations with exact *P* values to determine the association between the proportion of falls followed by a huddle and the repeat fall rate. At study end, we used the TeamSTEPPS® Teamwork Perceptions Questionnaire (T-TPQ) to assess perceptions of teamwork support for fall-risk reduction and the Hospital Survey on Patient Safety Culture (HSOPS) to assess perceptions of safety culture. We added an item to the T-TPQ for respondents to indicate the number of post-fall huddles in which they had participated. We used a binary logistic regression with a logit link to examine the effect of participation in post-fall huddles on respondent-level percent positive T-TPQ and HSOPS scores. We accounted for clustering of respondents within hospitals with random effects using the GLIMMIX procedure in SAS/STAT.

**Result:**

Repeat fall rates were negatively associated with the proportion of falls followed by a huddle. As compared to hospital staff who did not participate in huddles, those who participated in huddles had more positive perceptions of four domains of safety culture and how team structure, team leadership, and situation monitoring supported fall-risk reduction.

**Conclusions:**

Post-fall huddles may reduce the risk of repeat falls. Staff who participate in post-fall huddles are likely to have positive perceptions of teamwork support for fall-risk reduction and safety culture because huddles are a team-based approach to reporting, adapting, and learning.

**Electronic supplementary material:**

The online version of this article (10.1186/s12913-019-4453-y) contains supplementary material, which is available to authorized users.

## Background

It is estimated that 3% of hospitalized patients fall annually [1] and that approximately one-fourth of these falls result in injury [2] with associated excess costs of $7000 per injury [3]. Consequently, the U.S. Centers for Medicare and Medicaid Services (CMS) has categorized serious fall-related injuries as a preventable hospital-acquired condition (HAC) since 2008 [4]. Currently, there are 14 HACs for which the CMS no longer reimburses hospitals if the condition was not present on admission [4]. The complexity of falls as a patient safety problem is illustrated by the fact that a singular focus on either individual processes [5–7] or incentivizing outcomes such as non-payment for serious fall-related injuries has not significantly decreased the incidence of this HAC [8]. Thus, falls among hospital patients are a complex, “wicked” problem.

Wicked problems are by definition persistent, context-dependent, and lack definitive solutions [9]. Patient falls are complex because they result from a combination of patient (e.g. lower extremity weakness) [1, 2, 10, 11], environmental (e.g. tripping hazards) [12], and system factors. System factors that contribute to patient falls include the attitude that falls are inevitable [13], poor teamwork [14], and an inability to adequately learn from fall events [15]. Due to their complexity, wicked problems are best addressed using a sociotechnical, “systems” approach, which requires people to make sense of the multiple social and technical factors that contribute to the complex problem [16].

After-action reviews (AARs)—also referred to as debriefs and huddles—are a specific type of meeting that provides the opportunity for the collective sensemaking needed to address wicked problems [17]. A meta-analysis revealed that effective AARs may improve team performance by 25% through retrospective learning as team members make sense of an event to improve future performance [18]. This sensemaking requires a psychologically safe environment, which is most likely to occur when a facilitator guides the team to discuss what went well, what went poorly, what almost went poorly, and what will be done differently moving forward [19].

### Debriefs/huddles in healthcare

Because AARs have been implemented across multiple disciplines (e.g. military, aviation, law enforcement, first responders, and healthcare) and organizations there is ambiguity in the terms used to describe these sensemaking team meetings [20]. Team Strategies and Tools to Enhance Performance and Patient Safety (TeamSTEPPS), the national standard for team training in healthcare, defines debriefs and huddles as leadership tools. A debrief is defined as a tool to review a team’s performance by identifying key events (e.g. a patient fall), discussing what went well and what did not go well, identifying lessons learned, and planning to apply these lessons. A huddle is defined as a tool for communicating changes in a plan of care that are needed due to changes in the patient’s status (e.g. a fall) or because the current plan is not effective (e.g. interventions failed to prevent a fall) [21]. Reflecting the overlap in these definitions, it is common for healthcare professionals to refer to any post-event team meeting as a huddle.

Debriefing for learning and sensemaking is common in healthcare education and practice. Simulation-based research indicates that debriefing can improve participant knowledge, skill, and patient outcomes [22]. Additionally, debriefing after life-threatening emergencies was found to improve clinician satisfaction, technical and non-technical performance, and short-term patient outcomes [23]. Healthcare professionals and organizations such as the Veterans Administration [24], the Agency for Healthcare Research and Quality (AHRQ) [25], the Institute for Healthcare Improvement [26], and The Joint Commission [27] use the term post-fall huddle to describe the sensemaking component of an evidence-based fall-risk-reduction program [24, 25]. The post-fall huddle is intended to be an interdisciplinary team-driven process [28] to identify the causes of a patient fall and develop a plan to prevent a repeat fall. This process includes collecting information from the patient, family, and staff about what the patient was intending to do; the location of the fall; how the fall was discovered; the severity of any patient injury; the interventions intended to be in place; and changes in the plan of care needed to reduce the risk of another fall [25, 29]. Information collected from post-fall huddles should be aggregated and shared across the system [28].

Studies regarding the implementation of post-fall huddles are limited. Quigley and colleagues [24] implemented post-fall huddles as part of a multifactorial fall-risk-reduction strategy in a nine-hospital collaborative. Hoke and colleagues [30] did so in a cardiac care unit. Quigley et al. did not report the impact of conducting post-fall huddles on the study outcomes of decreasing the risk of falls and fall-related injury. Hoke et al. concluded that post-fall huddles may contribute to decreases in the incidence of falls and fall-related injury because they create a culture of reflection and open communication. However, they did not provide empirical evidence for this conclusion. Despite the call to implement post-fall huddles, to our knowledge, the effect of post-fall huddles on the relevant patient outcome of repeat falls has not been empirically tested.

### Debriefs/huddles and safety culture

Safety culture represents the learned, shared, and enduring assumptions, values, beliefs, and behaviors of staff regarding the organization’s willingness to detect and learn from errors [31, 32]. Our guiding theory views safety culture as an element of organizational context that moderates the effectiveness of patient safety practices [33]. Reason describes four categories of practices that exist within a culture of safety: (1) reporting of adverse events and near misses, (2) responding in a just and fair manner to individuals involved in adverse events, (3) adapting to changing circumstances using team skills such as structured communication, and (4) learning from experience [34].

Research reveals that specific interventions may improve perceptions of safety culture. A pre-post evaluation reported that the implementation of postoperative debriefs resulted in significant improvement in the perception of operating room safety culture among neurosurgeons, anesthesiologists, and nurses [35]. A systematic review found that team training, executive walkrounds, and the Comprehensive Unit-Based Safety Program are practices that may improve perceptions of safety culture [33]. Furthermore, a longitudinal study revealed that adoption of team behaviors led to transformational change in staff perceptions of all four categories of safety culture practices [36]. Because post-fall huddles are a patient safety practice that facilitates reporting, adapting, and learning in a just and fair systems-focused process, participation in huddles may affect perceptions of safety culture [37].

As a moderating contextual factor [33], safety culture is not a structure or process of care causally linked to clinical outcomes [38, 39]. Consequently, it is not surprising that empirical evidence seeking independent associations between safety culture and patient outcomes is mixed. Specifically, there are studies that have reported independent associations between safety culture and patient outcomes [40–43], and there are studies that have not reported independent associations [44, 45]. However, because of its influence on organizational structures and processes [39], developing a strong safety culture is a consistent recommendation for healthcare organizations [46, 47].

### Debriefs/huddles and perceptions of teamwork

The complexity of healthcare requires coordinated action within and between multiple teams to achieve a collective goal such as decreasing fall risk. A multiteam system (MTS) consists of two or more component teams that interact to achieve such a collective goal [48]. A typical healthcare MTS consists of at least three component teams: the core team that provides direct patient care, contingency teams made up of core team members who manage emergent events and conduct debriefs and huddles, and the coordinating team that manages component team performance to achieve specific goals [49]. Effective coordination across component teams achieves system goals such as decreasing fall risk by planning, standardizing, and adjusting processes in real time [50, 51]. In the MTS approach to fall-risk reduction, staff participating in post-fall huddles function as a contingency team that adjusts processes in real time to adaptively manage fall risk. Thus, participation in post-fall huddles may affect perceptions of teamwork.

In summary, conducting post-fall huddles is considered an integral component of a fall-risk-reduction program [24, 25]. However, to our knowledge, there is no empirical evidence linking post-fall huddles to patient outcomes or perceptions of teamwork and safety culture. The purpose of this paper is to determine the association between: (1) conducting a post-fall huddle and the risk of a repeat fall, (2) participating in a post-fall huddle and perceptions of teamwork support for fall-risk reduction, and (3) participating in a post-fall huddle and perceptions of patient safety culture. This study was approved by the University of Nebraska Medical Center Institutional Review Board (PROTOCOL # 256–12-EP).

## Methods

### Sample and procedure

This study included a longitudinal assessment of repeat fall rates and cross-sectional assessments of teamwork support for fall-risk reduction and safety culture. From August 2012 to July 2014, 16 small rural hospitals (Table 1) in the central U.S. participated in a research demonstration and dissemination study funded by AHRQ. The purpose of this funding mechanism was to: (1) implement safe practices that demonstrate evidence of reducing errors and risks associated with healthcare processes and (2) inform AHRQ, providers, patients, and payers about implementation of safe practices in diverse settings such as small rural hospitals, which care for a high proportion of older adults. An additional Excel file contains data describing hospital characteristics, numbers of falls, numbers of unique patients who fell, and numbers of post-fall huddles conducted. (See Additional file 1).

**Table 1:** Characteristics of falls and post-fall huddles among 16 hospitals

Hospital bed size, mean (SD)	26 (6)
Total number of falls (Range across 16 hospitals)	347 (5–49)
Total number of unique patients who fell (Range across 16 hospitals)	308 (4–43)
Total number of falls followed by a post-fall huddle	223
Total proportion of falls followed by a post-fall huddle (Range across 16 hospitals)	0.64 (0.29–0.96)
Repeat fall rate, mean (Range)	1.12 (1.00–1.45)

The purpose of our study, Collaboration and Proactive Teamwork Used to Reduce (CAPTURE) Falls, [51] was to decrease the risk of falls in small rural hospitals by using an MTS to implement evidence-based fall-risk-reduction practices. Reflecting the complementary skills needed to mitigate the patient, environmental, and system sources of fall risk, we implemented interprofessional fall-risk-reduction coordinating teams to lead the intervention in each hospital. These teams included at a minimum staff from nursing, pharmacy, physical and/or occupational therapy, and patient safety/quality improvement. We also developed a system for them to report, benchmark, and learn from fall events because lack of such a system contributes to fall risk [15]. The results of this study revealed that the more effectively hospitals used interprofessional teams to coordinate fall-risk-reduction structures and processes, the lower were their unassisted and injurious fall rates [51]. A requirement for inclusion in the study was that each hospital had previously implemented team strategies and tools consistent with the Team Strategies and Tools to Enhance Performance and Patient Safety (TeamSTEPPS®) curriculum [36].

#### The post-fall huddle intervention

**Fig. 1:**
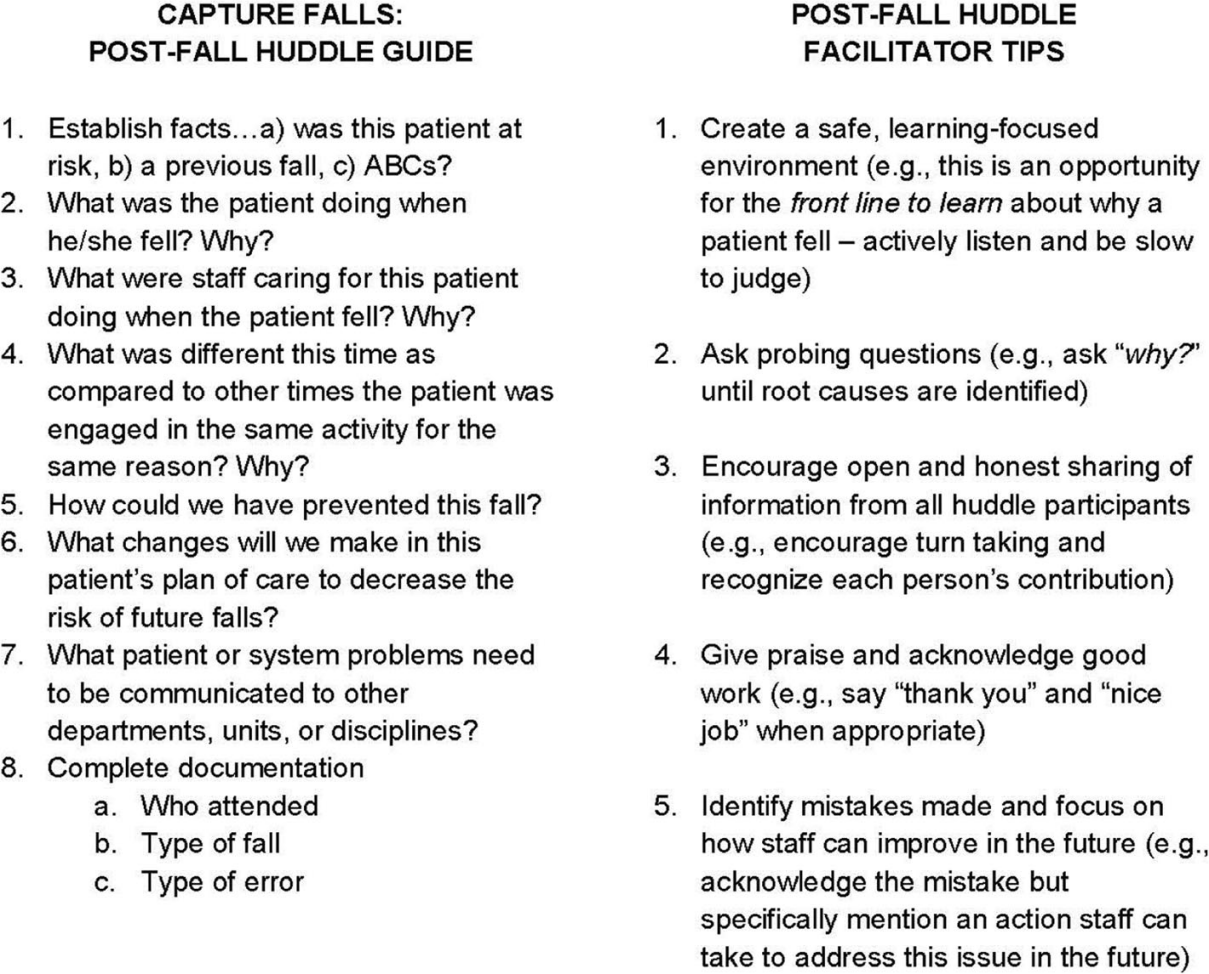
Post-Fall Huddle Pocket Guide

**Fig. 2:**
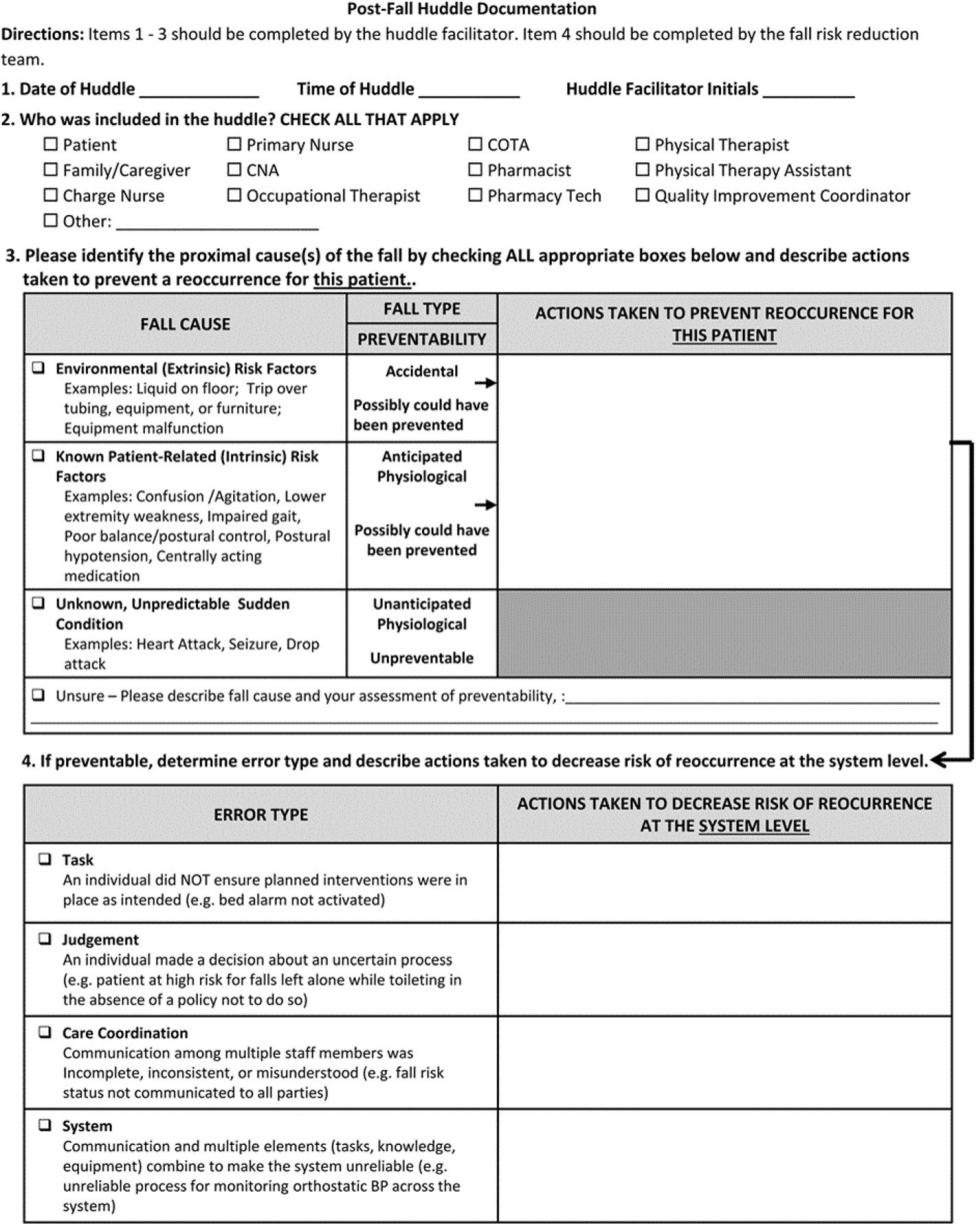
Post-Fall Huddle Documentation Form

We trained hospitals to conduct post-fall huddles for the purposes of: (1) sensemaking about the patient, environment, and system factors that contributed to a particular patient’s fall and to plan immediate actions to decrease the risk of a repeat fall [28]; (2) applying what was learned from a particular fall to the system [28]; and, (3) improving trust and team orientation among post-fall huddle participants [52]. We developed an online post-fall huddle training program, which includes a video demonstrating how to facilitate and participate in a huddle, a post-fall huddle pocket guide (Fig. 1), and a post-fall huddle documentation form (Fig. 2) [53].

The post-fall huddle pocket guide (Fig. 1) is a set of structured questions intended to establish psychological safety and facilitate a sensemaking conversation among staff providing care for the patient, members of the coordinating team available to attend the huddle, and patients/families. The facilitator completed the documentation form (Fig. 2) after the huddle to clarify the cause of the fall, errors associated with potentially preventable falls, and actions needed to prevent a future fall for the patient and to decrease the risk of a similar fall across the system. Categorizing the cause of a fall as preventable (anticipated physiological or accidental) or nonpreventable (unanticipated physiological) [54] supports sensemaking to decrease repeat falls [55]. Because about 85% of falls are preventable [54], we prompted huddle facilitators and the coordinating team to consider four types of organizational errors (task, judgement, coordination, and system) [56] that may have contributed to preventable falls and that should be addressed by the MTS in post-huddle actions. We reviewed the accuracy of post-fall huddle documentation with coordinating team members during quarterly conference calls to ensure errors received appropriate follow-up at the system level [28].

### Measures

#### Repeat fall rate

To calculate a hospital’s repeat fall rate, we divided the total number of falls reported by the hospital during the study by the number of unique medical record numbers associated with each fall. Thus, the aggregate mean repeat fall rate of 1.12 indicates that each patient who fell had a 12% chance of a repeat fall (Table 1). Repeat falls tend to be excluded as an outcome in fall-risk-reduction research [55], but are the patient-level outcome of interest in assessing the effectiveness of post-fall huddles. Standardizing the rate of repeat falls supports evaluation of interventions intended to prevent these falls and benchmarking of this outcome within and across hospitals [57].

#### TeamSTEPPS® teamwork perceptions questionnaire (T-TPQ)

The T-TPQ was developed by AHRQ to measure individuals’ perceptions of team skills and behaviors as taught in the TeamSTEPPS® team training curriculum [58]. It consists of 35 items distributed across five dimensions as presented in Table 2, which respondents rate using a 5-point Likert-type scale. We adapted the survey to elicit respondent perceptions about the use of teamwork to support fall-risk reduction (e.g., “Staff correct each other’s mistakes to ensure that *fall-risk-reduction* procedures are followed properly”). We added an item for respondents to indicate the number of post-fall huddles in which they had participated during the past two years (i.e., the duration of the CAPTURE Falls study). We used these responses to create two groups: those respondents who had participated in one or more post-fall huddles, and those who had not participated in a post-fall huddle.

**Table 2:** TeamSTEPPS® Teamwork Perceptions Questionnaire Percent Positive Scores by Post-Fall Huddle Participation

*Dimensions and Items*	*Post-Fall Huddle Participation*		*p value*
	*Yes* *(n varies 256 to 266)* ^*a*^	*No* *(n varies 440 to 472)* ^*a*^	
Team Structure (α = .92)	92	90	.63
1. The skills of all hospital staff overlap sufficiently so that work related to fall-risk-reduction can be shared when necessary.	92	91	.62
2. All hospital staff are held accountable for their actions related to fall-risk reduction.	87	89	.49
3. Staff within my unit/department share information that enables timely decision making about fall-risk reduction by the direct patient care team.	95	89	**.009**
4. My unit/department makes efficient use of resources related to fall-risk reduction (e.g., staff, supplies, equipment, information).	94	92	.37
5. Staff within my unit/department understand their roles and responsibilities related to fall-risk reduction.	95	95	.77
6. My unit/department has clearly articulated goals for fall-risk reduction.	93	86	**.003**
7. My unit/department operates at a high level of efficiency when it comes to fall-risk reduction.	91	88	.29
Leadership (α = .96)	91	82	**<.001**
1. My supervisor/manager considers staff input when making decisions about fall-risk reduction.	93	86	**.01**
2. My supervisor/manager provides opportunities to discuss the unit/department’s performance after a patient fall.	91	78	**<.001**
3. My supervisor/manager takes time to meet with staff to discuss the fall-risk-reduction program.	88	74	**<.001**
4. My supervisor/manager ensures that adequate resources (e.g., staff, supplies, equipment, information) are available to support the fall-risk-reduction program.	92	88	**.09**
5. My supervisor/manager successfully resolves conflicts involving the fall-risk-reduction program.	87	81	**.04**
6. My supervisor/manager models appropriate team behavior in support of the fall-risk-reduction program.	92	87	**.06**
7. My supervisor/manager ensures that staff are aware of any situations or changes that may affect the fall-risk-reduction program.	91	83	**.004**
Situation Monitoring (α = .89)	90	87	.26
1. Staff effectively anticipate each other’s needs when implementing fall-risk-reduction interventions.	92	88	**.08**
2. Staff monitor each other’s performance when implementing fall-risk-reduction interventions.	84	82	.60
3. Staff exchange relevant information to decrease the risk of falls as it becomes available.	94	91	**.08**
4. Staff continuously scan the environment for important information to decrease the risk of falls.	93	90	**.02**
5. Staff share information regarding potential complications that may increase a patient’s risk of falls (e.g., change in status, previous fall).	95	91	**.07**
6. Staff meet to reevaluate a patient’s fall-risk-reduction plan of care when aspects of the situation have changed.	88	82	**.049**
7. Staff correct each other’s mistakes to ensure that fall-risk-reduction procedures are followed properly.	84	84	.96
Mutual Support (α = .92)	89	87	.42
1. Staff assist fellow staff to decrease the risk of falls during a high workload.	93	91	.24
2. Staff request assistance from fellow staff to implement fall-risk-reduction interventions when they feel overwhelmed.	91	93	.47
3. Staff caution each other about potentially dangerous situations that may increase the risk of patient falls.	94	93	.54
4. Feedback between staff about fall-risk reduction is delivered in a way that promotes positive interactions and future change.	90	88	.30
5. Staff advocate for patients who are at risk for falls even when their opinion conflicts with that of a senior member of the unit/department.	90	90	.98
6. When staff have a concern about a patient’s risk of falling, they challenge others until they are sure the concern has been heard.	84	80	.24
7. Staff resolve their conflicts about fall-risk reduction, even when the conflicts have become personal.	82	76	**.07**
Communication (α = .94)	92	90	.24
1. Information about fall-risk reduction is explained to patients and their families in lay terms.	95	91	**.06**
2. Staff relay relevant information about fall-risk reduction in a timely manner.	95	92	.18
3. When communicating with patients about fall-risk reduction, staff allow enough time for questions.	93	92	.63
4. Staff use common terminology when communicating with each other about fall-risk reduction.	96	94	.15
5. Staff verbally verify information about a patient’s fall risk that they receive from each other.	93	90	.23
6. Staff follow a standardized method of sharing fall risk information when handing off patients.	89	87	.44
7. Staff seek fall-risk-reduction information from all available sources.	84	85	.96

Bold *p* values indicate differences between groups that are statistically significant at *p* < .05 or of interest with *p* < .10

^a^Number of respondents varies for each dimension due to the requirement to complete at least five items to calculate the dimension percent positive score

#### Hospital survey on patient safety culture (HSOPS)

The HSOPS is a psychometrically sound [59] instrument developed by AHRQ to provide healthcare organizations with a valid tool to assess hospital safety culture. It consists of 42 items distributed across 12 dimensions as presented in Table 3, which respondents rate using a 5-point Likert-type scale. Nine of the 12 dimensions assess safety culture at the unit/department level and the remaining three dimensions assess safety culture at the level of the hospital as a whole.

**Table 3:** Hospital Survey on Patient Safety Culture percent positive scores by post-fall huddle participation

*Dimensions and Items*	*Post-Fall Huddle Participation*		*p Value*
	*Yes* *(n varies 218 to 221)* ^*a*^	*No* *(n varies 357 to 368)* ^*a*^	
Overall perception of Safety (α = .92)	76	76	.83
1. Patient safety is never sacrificed to get more work done.	72	75	.50
2. Our procedures and systems are good at preventing errors from happening.	82	79	.40
3. It is just by chance that more serious mistakes don’t happen around here.^b^	76	71	.16
4. We have patient safety problems in this department.^b^	75	79	.27
Frequency of Events Reported (α = .97)	70	66	.48
1. When a mistake is made, but is caught and corrected before affecting the patient, how often is this reported?	58	58	.93
2. When a mistake is made, but has no potential to harm the patient, how often is this reported?	70	63	**.09**
3. When a mistake is made that could harm the patient, but does not, how often is this reported?	81	77	.17
Supervisor/Manager Expectations & Actions Promoting Patient Safety (α = .92)	83	80	.88
1. My supervisor/manager says a good word when he/she sees a job done according to established patient safety procedures.	73	74	.70
2. My supervisor/manager seriously considers staff suggestions for improving patient safety.	85	81	.25
3. Whenever pressure builds up, my supervisor/ manager wants us to work faster, even if it means taking shortcuts.^b^	88	83	**.10**
4. My supervisor/manager overlooks patient safety problems that happen over and over.^b^	84	82	.43
Organizational Learning—Continuous Improvement (α = .86)	85	79	**.10**
1. We are actively doing things to improve patient safety.	96	91	**.03**
2. Mistakes have led to positive changes here.	77	71	**.08**
3. After we make changes to improve patient safety, we evaluate their effectiveness.	83	74	**.01**
Teamwork Within Departments (α = .92)	87	85	.63
1. People support one another in this department.	91	92	.80
2. When a lot of work needs to be done quickly, we work together as a team to get the work done.	94	94	.94
3. In this department, people treat each other with respect.	85	81	.17
4. When one area in this department gets really busy, others help out.	77	74	.35
Communication Openness (α = .90)	64	63	.88
1. Staff will freely speak up if they see something that may negatively affect patient care.	78	79	.89
2. Staff feel free to question the decisions or actions of those with more authority.	52	46	.16
3. Staff are afraid to ask questions when something does not seem right.^b^	63	64	.74
Feedback and Communication About Error (α = .84)	69	68	.71
1. We are given feedback about changes put into place based on event reports.	61	56	.27
2. We are informed about errors that happen in this department.	68	71	.50
3. In this department, we discuss ways to prevent errors from happening again.	79	78	.69
Nonpunitive Response to Error (α = .87)	64	56	**.05**
1. Staff feel like their mistakes are held against them.^b^	70	63	**.07**
2. When an event is reported, it feels like the person is being written up, not the problem.^b^	69	56	**<.001**
3. Staff worry that mistakes they make are kept in their personnel file.^b^	54	49	.17
Staffing (α = .96)	73	69	.31
1. We have enough staff to handle the workload.	76	70	.14
2. Staff in this department work longer hours than is best for patient care.^b^	61	58	.59
3. We use more agency/temporary staff than is best for patient care.^b^	80	78	.52
4. We work in “crisis mode” trying to do too much, too quickly.^b^	73	68	.27
Hospital Management Support for Patient Safety (α = .92)	83	80	**.10**
1. Hospital management provides a work climate that promotes patient safety.	93	89	.13
2. The actions of hospital management show that patient safety is a top priority.	83	81	.48
3. Hospital management seems interested in patient safety only after an adverse event happens.^b^	73	69	.35
Teamwork Across Hospital Departments (α = .88)	75	66	**.011**
1. There is good cooperation among hospital departments that need to work together.	76	67	**.02**
2. Hospital departments work well together to provide the best care for patients.	86	76	**.003**
3. Hospital departments do not coordinate well with each other.^b^	62	52	**.02**
4. It is often unpleasant to work with staff from other hospital departments.^b^	77	67	**.01**
Hospital Handoffs and Transitions (α = .96)	61	52	**.07**
1. Things “fall between the cracks” when transferring patients from one department to another.^b^	59	50	**.04**
2. Important patient care information is often lost during shift changes.^b^	63	50	**.003**
3. Problems often occur in the exchange of information across hospital departments.^b^	60	50	**.03**
4. Shift changes are problematic for patients in this hospital.^b^	63	57	.15

Bold *P* values indicate differences between groups that are statistically significant at p < .05 or of interest with *p* ≤ .10

^a^Number of respondents varies for each dimension due to the requirement to complete at least three items to calculate the dimension percent positive score

^b^Reverse-worded item

In February and March 2014, we invited 2771 staff across the 16 hospitals to complete an electronic version of the HSOPS. Consistent with the survey user’s guide [60], these staff included: staff who provided direct patient care, those whose work directly affected patient care, providers, and administrators/managers. In June through August 2014, we invited 1649 staff across the 16 hospitals to complete the electronic, adapted version of the T-TPQ. Consistent with the survey manual [58] these staff included: those who provided direct patient care, provided services in patient rooms, were members of the fall-risk-reduction coordinating team, or were administrators/managers. We used the Dillman tailored-design methodology to maximize the response rate for both surveys [61]. An additional Excel file contains data from the T-TPQ and HSOPS surveys. (See Additional file 2).

### Analysis

We used SAS/STAT software, Version 9.4 (© 2002–2012) of the SAS System for Windows (SAS Institute Inc., Cary, NC, USA) to conduct all analyses. We used Spearman correlations with exact *P* values to determine the association between the proportion of falls that were followed by a post-fall huddle and the repeat fall rate for each of the 16 hospitals. We calculated percent positive scores for the T-TPQ and HSOPS as recommended in the manual or user’s guide for each survey [58, 60]. We calculated dimension scores for the T-TPQ when a respondent had completed at least five of seven items in a dimension. We calculated dimension scores for the HSOPS when a respondent had completed at least three items in a dimension. We calculated Cronbach’s alpha for each dimension in both surveys to ensure adequate internal consistency of the dimensions in our sample. We used a binary logistic regression with a logit link to examine the effect of participation in post-fall huddles on the percent positive T-TPQ and HSOPS scores at the level of the respondent. We accounted for clustering of respondents within hospitals with random effects using the GLIMMIX procedure. All statistical tests were two-sided. We considered probability values less than .05 statistically significant, and those equal to or less than .10 of interest. The datasets supporting the conclusions of this article are included within the article (and its additional files).

## Results

### Association between post-fall huddle prevalence and repeat fall rates

**Fig. 3:**
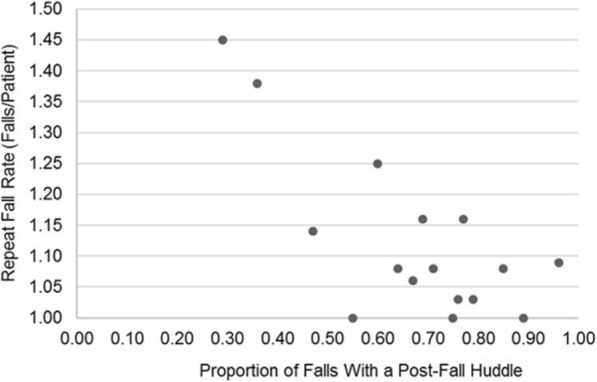
Association between Post-Fall Huddles and Repeat Fall Rates

Among the 16 hospitals, 308 unique patients experienced 347 falls, 64% of which were followed by a post-fall huddle (Table 1). Figure 3 illustrates the negative association between the proportion of falls within each hospital that were followed by a post-fall huddle and the repeat fall rate for that hospital. Specifically, the Spearman rank correlation coefficient was −.47 (*p* = .07), which is considered a moderate effect size.

### Association between post-fall huddle participation and perceptions of teamwork (Table 2)

The aggregate T-TPQ response rate was 49.4% (814/1649), ranging from 36 to 75% among the 16 hospitals. Cronbach’s alpha for each of the five dimensions ranged from .89 to .96, indicating adequate internal consistency of the customized items. Approximately one-third (266) of respondents indicated that they had participated in at least one post-fall huddle. In general, staff perceptions of teamwork were consistently high such that percent positive scores exceeded 80% for nearly all items regardless of participation in a post-fall huddle. However, as compared to those respondents who did not participate in post-fall huddles, those who did participate in at least one huddle had significantly more positive perceptions of:

- two of seven items in the *Team Structure* dimension (e.g., “My unit/department has clearly articulated goals for fall-risk reduction”).
- the *Team Leadership* dimension (e.g., “My supervisor/manager provides opportunities to discuss the unit/department’s performance after a patient fall”).
- two of seven items in the *Situation Monitoring* dimension (e.g., “Staff meet to reevaluate a patient’s fall-risk-reduction plan of care when aspects of the situation have changed”).

### Association between post-fall huddle participation and perceptions of safety culture (Table 3)

The aggregate response rate for the HSOPS was 66.8% (1843/2761), ranging from 40 to 84% across the 16 hospitals. Cronbach’s alpha for each of the 12 dimensions ranged from .84 to .97, indicating adequate internal consistency. As compared to those respondents who did not participate in post-fall huddles, those who did participate in at least one huddle had significantly more positive perceptions of:

- two of three items in the *Organizational Learning* dimension (e.g. “After we make changes to improve patient safety, we evaluate their effectiveness”),
- one reverse-worded item within the *Nonpunitive Response to Error* dimension (e.g. “When an event is reported, it feels like the person is being written up, not the problem”),
- the *Teamwork Across Hospital Departments* dimension (e.g. “Hospital departments work well together to provide the best care for patients”), and
- three of four reverse-worded items within the *Hospital Handoffs and Transitions* dimension (e.g. “Important patient care information is often lost during shift changes”).

## Discussion

We sought to determine the association between conducting post-fall huddles and the risk of repeat falls, and we sought to determine the association between staff participation in post-fall huddles and their perceptions of teamwork support for fall-risk reduction and safety culture. Our approach was intended to address the lack of evidence linking the implementation of post-fall huddles as a sensemaking component of evidence-based fall-risk-reduction programs to patient outcomes and staff perceptions of teamwork and safety culture.

First, our results demonstrate that the greater the proportion of falls in a hospital that are followed by a post-fall huddle, the lower may be the repeat fall rate. Given our sample size of 16 hospitals, we believe this finding to be of interest despite the moderate effect size (*p* = .07). To our knowledge, these are the first empirical results to demonstrate that conducting post-fall huddles may achieve the intended goal of decreasing the risk of a repeat fall. These results are consistent with a previous meta-analysis that revealed that effective AARs may improve team performance by 25% [18].

Second, our results demonstrate that staff perceptions of teamwork were consistently high regardless of participation in a post-fall huddle. This finding may reflect the facts that all respondents were trained in teamwork and participated in a two-year quality improvement collaborative that sought to use MTSs to implement evidence-based fall-risk-reduction practices. However, those staff who participated in post-fall huddles had significantly more positive perceptions of items within three dimensions of the T-TPQ than did staff who did not participate in post-fall huddles. Those items and dimensions that were differentially impacted by participation in post-fall huddles reflected the sensemaking purpose of the huddle (i.e. information sharing, goal setting, scanning the environment, and re-evaluating the plan of care after a change) and our training which emphasized effective facilitation of the huddle as a team leadership behavior. To our knowledge, these results are the first to use the T-TPQ to measure the impact of participating in a structured team meeting (i.e. post-fall huddle) on perceptions of teamwork.

Third, our results demonstrate that staff perceptions of items within four dimensions of safety culture were also differentially impacted by participation in post-fall huddles. These dimensions—*Organizational Learning, Nonpunitive Response to Error, Teamwork Across Hospital Departments,* and *Hospital Handoffs and Transitions—*reflect our implementation of the post-fall huddle as an interdisciplinary team-based approach to organizational learning in which a facilitator establishes an atmosphere of psychological safety and participants leave with a plan. This plan may address fall-risk factors and interventions that “fell through the cracks” when patients were transferred across departments, and it may address information that was lost during shift changes. These results are consistent with studies that have reported improved perceptions of safety culture in association with the adoption of structured team strategies and tools [33, 35, 36]. Finally, our results are consistent with previous research describing the positive impact of AARs on outcomes in healthcare [22, 23] and other contexts [19].

### Limitations

This study has limitations. First, our sample size was limited to 16 hospitals due to the resource-intense participatory nature of the study. Second, our three measures were self-reported by hospital staff. However, the three measures triangulate to reveal the impact of post-fall huddles on patient and staff outcomes. In reality, post-fall huddles are conducted in response to voluntarily reported falls, and voluntary reporting of falls is the standard of practice for fall-related quality improvement and benchmarking [62]. Relying on voluntary reporting of falls is consistent with the goal of maximizing external generalizability of our demonstration study. Furthermore, measures of culture are by nature self-report as they seek to evaluate respondents’ perceptions. Finally, the MTS focus of the study may have created a ceiling effect for exploring group differences based on post-fall huddle participation. Consequently, differences between groups at the dimension (e.g. *Hospital Management Support for Patient Safety*) and item level (e.g. “*Information about fall-risk reduction is explained to patients and their families in lay terms.”*) with *p* values equal to and between .05 and .10 are of interest because they are consistent with the structure, process, and outcomes of post-fall huddles.

### Strengths, practical implications and future research

This study has strengths and implications for practice and research. Strengths include our detailed description of our post-fall huddle intervention, survey response rates, definitions of key concepts and statement of a guiding theory [63]. The detailed description of our intervention (i.e. the online training program, pocket guide, and documentation form that facilitates categorization of organizational errors) is consistent with a recent call for improved description of the implementation of fall-risk-reduction interventions in published studies [64]. We conceptualized safety culture as a contextual element that moderates the effectiveness of patient safety practices [33, 39]. In addition, there is consistency between the unit of analysis of our intervention (individual staff participation in a post-fall huddle) and our culture assessments (individual T-TPQ and HSOPS percent positive scores) [65]. Furthermore, because we were specifically interested in perceptions of teamwork support for fall-risk-reduction, which reflects the nature of our post-fall huddle intervention, we customized the T-TPQ to assess this specific construct. Our results support the use of a structured program to train staff to facilitate and document post-fall huddles and the allocation of resources to ensure staff, patients, and families participate in post-fall huddles because the benefits of doing so appear to outweigh the risks [66]. The benefits include decreased risk of repeat falls and improved perceptions of teamwork and safety culture. The risks would be staff time in training and conducting poor quality huddles that waste staff time.

Additional research is needed to determine whether our post-fall huddle training is scalable to larger hospitals and yields similar results. Second, we need to determine whether our results may underestimate the impact of participation in post-fall huddles on perceptions of teamwork and safety culture in hospitals naïve to team training and the MTS approach to fall-risk reduction. Finally, future research should explore whether desired outcomes vary according to participants in the huddles and the quality of facilitation. Given the impact of post-fall huddle participation on perceptions of *Teamwork Across Hospital Departments*, further research should compare the effectiveness of interdisciplinary post-fall huddles including physical/occupational therapists and pharmacists to nursing only huddles. The effectiveness of post-fall huddles is likely to vary since consistently conducting huddles in a structured format, which seems necessary to produce desired outcomes [67], requires training and dedicated resources.

Our approach and results contribute to safety culture research in general. First, they may help explain why some studies [44, 45] did not find independent associations between safety culture and patient outcomes. We conceptualized safety culture as an element of the context [33] in which our collaborative hospitals delivered care and not as part of the causal path that produces patient outcomes [38]. Conceptually, training staff to effectively lead post-fall huddles improved the structure of care; actions taken as a result of the huddles affected the process of care, which in turn decreased the incidence of repeat falls. Staff exposure to this causal path likely influenced their perceptions of teamwork and safety culture [39].

Second, our results support the call to report assessments of safety culture at the item level [65] because each item within a dimension may measure a slightly different aspect of a complex phenomenon such as teamwork or safety culture. If we had restricted our interest to significant differences between groups at the dimension level and ignored differences at the item level we would have wrongly concluded that participation in post-fall huddles is not associated with important aspects of *Team Structure, Situation Monitoring, Organizational Learning*, *Nonpunitive Response to Error* and *Hospital Handoffs and Transitions* that reflect the structure and process of post-fall huddles. For example, the reverse-worded item within *Nonpunitive Response to Error* that is significantly more positive for those participating in post-fall huddles, *“When an event is reported, it feels like the person is being written up, not the problem,”* reflects the fact that effective post-fall huddles provide a nonpunitive, psychologically safe process to focus on the problem and not the person. This approach to analyzing survey results was used in previous research to link changes in the structure of care (team training) with perceptions of safety culture at the item level [36]. Further, this approach is consistent with Cronbach and Gleser’s [68] work, which suggests that broad measures predict broad criteria with moderate validity while maximum validity requires a high degree of fidelity between the measure and the criterion. Specifically, a narrow measure such as an item is more likely to accurately depict the essential characteristics of the criterion of interest [69] (e.g. psychological safety) as illustrated above.

## Conclusions

Post-fall huddles improve the capacity of small rural hospitals to make sense of the wicked problem of patient falls and thus decrease the risk of a repeat fall. Staff participating in post-fall huddles function as a contingency team that improves coordination of the fall-risk-reduction MTS by adjusting fall-risk-reduction processes in real time. Thus, post-fall huddles can decrease the repeat fall rate, which is an important patient safety outcome appropriate for benchmarking within and across hospitals. As an interdisciplinary team-based approach to learning and sensemaking, staff who participate in post-fall huddles are likely to have positive perceptions of teamwork support for fall-risk reduction and patient safety culture. When senior leaders commit the resources needed to implement and document effective post-fall huddles, staff may perceive that the organization is committed to learn from each fall and to continuously decrease the risk of patient falls across the system. Future research seeking associations between safety culture and patient outcomes should heed previous calls to define key concepts and ground their work in theoretical frameworks [63] that link measures of safety culture to measures of the structure and process of care at a consistent unit of analysis (e.g. individual, unit/department, or hospital) [65].

## Additional files

Additional file 1: This dataset contains 11 variables that provide information about the characteristics of the 16 hospitals participating in this study, the number of fall events each hospital reported during the study, the number of unique patients who fell, and the number of falls followed by a post-fall huddle. (XLSX 11 kb)

Additional file 2: This dataset contains two worksheets. One worksheet contains responses from hospital staff who completed the Hospital Survey on Patient Safety Culture (HSOPS) and the TeamSTEPPS® Teamwork Perceptions Questionnaire (T-TPQ). The second worksheet is a data dictionary. (XLSX 765 kb)

## Data Availability

The datasets supporting the conclusions of this article are included within the article and its additional files.

## Abbreviations

AARs: After-action reviews
AHRQ: Agency for Healthcare Research and Quality
CAPTURE: Collaboration and Proactive Teamwork Used to Reduce Falls
CMS: U.S. Centers for Medicare and Medicaid Services
HAC: Hospital-acquired condition
MTS: Multiteam system
TeamSTEPPS®: Team Strategies and Tools to Enhance Performance and Patient Safety
T-TPQ: TeamSTEPPS® Teamwork Perceptions Questionnaire; HSOPS, Hospital Survey on Patient Safety Culture
